# Editorial: The contribution of viruses and innate immune system in the pathogenesis of type 1 diabetes

**DOI:** 10.3389/fendo.2023.1335716

**Published:** 2023-12-15

**Authors:** Laura Nigi, Jutta E. Laiho, Heikki Hyöty, Francesco Dotta

**Affiliations:** ^1^ Department of Medical Sciences, Surgery and Neurosciences, University of Siena, Siena, Italy; ^2^ Department of Virology, Faculty of Medicine and Health Technology, Tampere University, Tampere, Finland

**Keywords:** type 1 diabetes, viruses, innate immunity, adaptive immunity, inflammation

Type 1 diabetes (T1D) is a chronic autoimmune disease, characterized by the selective dysfunction and destruction of pancreatic β-cells, resulting in insulin deficiency (1). While the main culprits appear to be autoreactive CD8+ T lymphocytes, the (auto)immune response is likely more complex, encompassing cells from the adaptive as well as from the innate immune system. In this regard, the potential involvement of neutrophils, mast cells and NK cells in T1D pathogenesis has been spotlighted, revealing a scenario where β-cells, by recognizing immune cells and interacting with them, could serve as a connection linking the adaptive and innate immune systems, thereby playing a role in determining their own fate (2, 3). The incidence of T1D has risen over the past decades to an extent that underscores the influence of environmental factors in the disease’s development (4, 5). Accumulating evidence since the 1960’s has associated enterovirus infections, especially group B coxsackieviruses, with T1D development. Epidemiological studies have shown a heightened occurrence of T1D cases in the aftermath of enterovirus epidemics, and the presence of enterovirus RNA has been identified in the peripheral blood of new-onset T1D patients (6–8). In addition, enteroviruses have been identified in pancreatic islets of new-onset T1D patients (9) as well as of recent-onset T1D organ donors (10). Moreover, enterovirus capsid protein VP1 has been observed in pancreatic islets of T1D patients (9, 11). Enteroviruses exhibit a tropism to pancreatic islets and to β-cells (12), likely mediated by the Coxsackie- and adenovirus receptor strongly expressed in β-cells (13); accordingly, insulin-producing cells are vulnerable to enteroviral infections in vitro, leading to a spectrum of effects that span from compromised function to cell death (14). Indeed, enteroviruses have been demonstrated to exert cytolytic effects on β-cells, potentially exposing previously concealed self-components; furthermore some serotypes could replicate without overtly destroying insulin-producing cells, but at the same time impairing their function (15). Alternatively, damage to β-cells may stem from a virus-induced inflammatory response within the pancreas. Viral infections can elicit the release of proinflammatory cytokines and the activation of (endogenous) antigen-presenting cells (APCs), alongside or instead of direct tissue damage (16). The inflammation resulting from a virus infection can potentially lead to autoreactive T cells generation through mechanisms termed “bystander activation” or “molecular mimicry” or a combination of both processes (16). In individuals who cannot efficiently eliminate the virus, it may persist within β-cells in a low-level replication state, continuing to produce viral RNA and proteins, which in turn constantly stimulate the innate immune system, sustaining inflammation and autoimmune reactions. Drawing from this information, it’s conceivable that eradicating such a persistent low-grade infection could enhance β-cells’ function in T1D, as suggested by a phase 2 clinical trial designed as a randomized, double-blind, and placebo-controlled study (17). In this trial an antiviral treatment combining pleconaril and ribavirin was able to positively affect insulin production in new-onset T1D children and adolescents, contributing to the preservation of residual β-cells’ function. Another aspect to consider in T1D pathogenesis is the role of the pancreas as a whole, and not just the endocrine compartment ie. islets that make up only ~2% of the entire pancreas (18). Indeed, while pancreatic endocrine and exocrine compartments are typically viewed as distinct entities, they are inherently interconnected (19). Importantly, in recent studies enterovirus RNA has been detected both in islets and in exocrine tissue of individuals with T1D and those at risk for the disease (20). Given these observations, delving deeper into the potential active role of the pancreatic exocrine tissue in T1D development emerges as a compelling avenue for further research.

All in all, numerous facets remain unresolved concerning the interplay between viruses and innate immune system and subsequent effect of inflammation in T1D development. In this series of four articles, experts in the field delved into these pivotal questions. First of all Wang et al. strengthened the evidence of a link between enteroviral infections and the risk of T1D in an updated comprehensive meta-analysis of 38 case-control studies. Indeed, the authors’ findings show an association, evident in blood as well as in tissue samples, in various populations, including European, African, Asian, Australian and Latin American. Krogvold et al. identified antiviral tissue responses and increased cell stress within pancreatic islets of patients with newly diagnosed T1D from the DiViD cohort in a study whose the objective was to compare gene and protein expression of specific virus-induced pathogen recognition receptors and interferon-stimulated genes in islets of newly diagnosed T1D subjects, compared to age-matched non-diabetic controls. As such, these findings strengthen previous observations supporting the existence of a persistent low-grade enteroviral infection in pancreatic islets and reinforce the theory positioning enteroviruses as key players in T1D development. Liu et al. underscored the unique association of enteroviral infections with T1D, in a study evaluating the presence of enterovirus RNA in type 2 diabetes (T2D) organ donors’ pancreases and examining its correlation with disease progression. The authors showed that although both T1D and T2D display notable similarities in terms of inflammatory markers within islets, enteroviral infiltration over the long term represents a distinctive pathological feature of T1D and its associated autoimmunity. Finally, Välikangas et al. draw attention to the hypothesis that T1D could be considered a disease involving the whole pancreas and not just the endocrine component. The authors performed a comprehensive analysis of the whole-pancreas gene expression in new-onset T1D patients from the DiViD study compared to non-diabetic controls and observed a heightened expression of core acinar cell genes, which encode digestive enzymes, in the entire pancreas of DiViD patients. Specifically, an upregulation of inflammatory and immune response genes was observed in DiViD patients’ pancreatic islets in contrast to the whole pancreas. The highlighted features underscore that T1D progression is associated with concurrent alterations in both the exocrine and endocrine components of the pancreas, which in turn may induce an imbalance and impaired communication among different cell populations. Hence the importance to consider both pancreatic compartments for better understanding molecular mechanisms of T1D.

## Author contributions

LN: Writing – original draft. JL: Writing – original draft. HH: Writing – review & editing. FD: Writing – review & editing.
